# When Consumption Leads to Catastrophe: A Case of Recurrent Alcohol Withdrawal Catatonia in an Adult Patient

**DOI:** 10.7759/cureus.61301

**Published:** 2024-05-29

**Authors:** Yatika Chadha, Saket S Toshniwal, Ragini Patil

**Affiliations:** 1 Psychiatry, Jawaharlal Nehru Medical College, Datta Meghe Institute of Higher Education and Research, Wardha, IND; 2 General Medicine, Jawaharlal Nehru Medical College, Datta Meghe Institute of Higher Education and Research, Wardha, IND

**Keywords:** motor dysregulation syndrome, recurrent catatonia, alcohol withdrawal, alcohol, catatonia

## Abstract

Catatonia is a motor dysregulation syndrome and a multifaceted neuropsychiatric behavioral syndrome distinguished by abnormal movements, immobility, abnormal behavior, and withdrawal, where patients are unable to move normally despite full physical capacity. Catatonia, in the background of alcohol withdrawal, is a fairly rare phenomenon. Therefore, we are reporting a case where the patient has a history of binge alcohol consumption, with catatonia reoccurring with his withdrawal symptoms, and no other illicit drug use in his history. Its rarity, complex presentation, and potential diagnostic pitfalls necessitate heightened awareness among healthcare professionals.

## Introduction

Alcohol withdrawal syndrome (AWS) encompasses a spectrum of symptoms ranging from mild tremors to life-threatening seizures to delirium tremens. Among the myriad of manifestations, catatonia is a multifaceted neuropsychiatric behavioral syndrome distinguished by abnormal movements and behaviors, immobility, and withdrawal by motor dysregulation, which stands as a rare yet formidable complication [1]. The main symptoms include mutism (inability to speak), negativism (resistance to simple commands), posturing and rigidity (abnormal body positions), persistent staring, repetitive movements (often self-injurious), automatic obedience (responding to tactile stimuli despite instructions), and a lack of pain response [1].

Catatonia symptoms may often be confused with other psychological or neurological diseases, leading to delayed diagnosis and care [2]. While catatonia is most commonly associated with psychiatric disorders, with an incidence of 7-17%, its emergence in the context of alcohol withdrawal is even less common and presents a distinctive challenge for clinicians [2]. We present a case report detailing the recurrent episodes of alcohol withdrawal catatonia in a 29-year-old adult male with a history of chronic alcohol abuse. This case underscores the complexity of managing AWS and highlights the potential for catatonia to manifest as a recurrent complication, necessitating vigilant monitoring and tailored treatment approaches, through a comprehensive exploration of the clinical presentation, diagnostic considerations, and management strategies. In this case report, we aim to contribute to the growing understanding of this uncommon but clinically significant phenomenon.

## Case presentation

**Figure 1:**
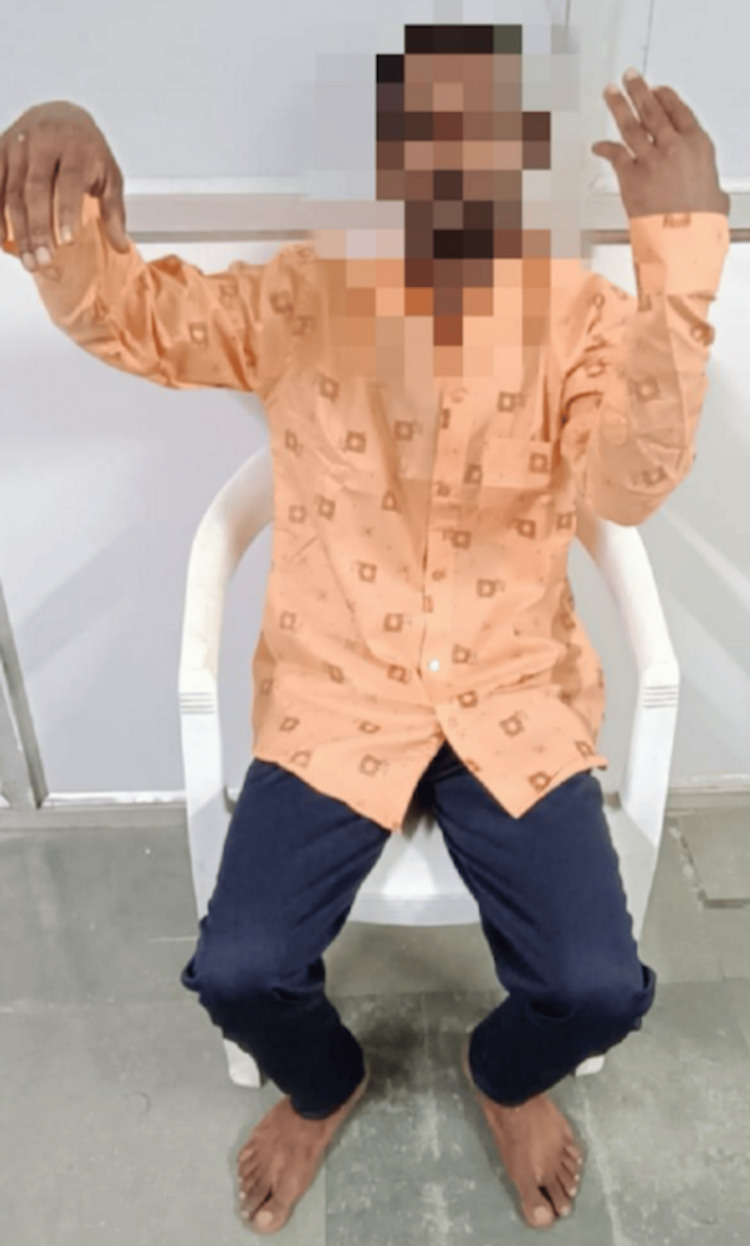
Catatonic posture of the patient

A 29-year-old man, a daily wage laborer by occupation with a history of alcohol use disorder presented with a pattern of binge drinking over the course of the last three years. His usual pattern is five to six consecutive days of binge drinking followed by a period of abstinence for two months. This time the patient had finished a five-day drinking binge, where he took approximately 700 mL of country-made liquor with the last intake being three days back as per the patient. The day before his current consultation he was brought in after exhibiting unusual behavior for a day, which included a lack of interaction, poor self-care, prolonged staring, and a fearful appearance. On examination, the patient revealed catatonic features such as mutism, staring, posturing, negativism, and ambitendency, but there were no overt signs of alcohol withdrawal. The catatonic posture of the patient is shown in Figure 1.

When questioned about his fearfulness and hallucinatory behavior, the patient stated that he had heard voices the day before but withheld any additional information. His routine blood investigations like complete blood count, liver function test, kidney function test, serum electrolytes, serum vitamin B12 levels, and thyroid profile were within normal limits. An electroencephalogram (EEG) and magnetic resonance imaging (MRI) of the brain were advised to rule out any organic cause, but unfortunately, the patient was not willing to do so. However, on applying the Bush-Francis Catatonia Rating Scale (BFCRS) for the severity of catatonia, he scored 29 points, as explained in Table 1.

**Table 1:** The Bush-Francis Catatonia rating scale score of 29 points for the patient

Sign	Score	Maximum score
Stupor	1	3
Mutism	1	3
Staring	2	3
Posturing	2	3
Grimacing	2	3
Echopraxia/echolalia	0	3
Stereotypy	2	3
Mannerisms	2	3
Stereotyped and meaningless repetition of words and phrases (verbigeration)	2	3
Rigidity	2	3
Negativism	2	3
Waxy flexibility	0	3
Withdrawal	2	3
Excitement	0	3
Impulsivity	0	3
Automatic obedience	0	3
Passive obedience	0	3
Muscle resistance	2	3
Ambitendency	3	3
Grasp reflex	0	3
Combativeness	0	3
Autonomic abnormality	2	3
Perseveration	2	3

The BFCRS consists of 23 items, where 13, and 17-21 are rated as either absent or present with a score of 0 or 3, respectively, while all other items are rated according to their severity on a scale from 0 to 3. On this scale, 0 indicates absence, 1 indicates occasional presence, 2 indicates frequent presence, and 3 indicates constant presence of the respective symptom. The BFCRS total score ranges from 0 to 69. The first 14 items of the BFCRS form the Bush-Francis Catatonia Screening Instrument (BFCSI). The BFCSI is used to assess the presence or absence of catatonia. As it was present in our case, it was diagnosed as catatonia. If two or more items on the BFCSI are rated as present, irrespective of their severity, catatonia is diagnosed. The interpretation of the BFCRS score is explained in Table 2. 

**Table 2:** BFCRS score interpretation BFCRS: Bush-Francis catatonia rating scale

BFCRS score range	Interpretation
0-7	Minimal to no catatonic symptoms
8-14	Mild catatonia, some symptoms present
15-21	Moderate catatonia, significant impairment
22 and above	Severe catatonia, marked disturbances

Our patient scored a total of 29 points, which falls under the severe catatonia category according to the BFCRS. The Clinical Institute Withdrawal Assessment (CIWA) score of the patient was 13 suggesting moderate withdrawal. The patient had experienced similar symptoms such as fearfulness, posturing, and mutism at the termination of at least four prior binge episodes, as reported by the informant. Each time, these symptoms were resolved within a day or two after treatment by a general practitioner. Past history indicated treatment from a government hospital, with records showing tablet olanzapine 10 mg once at night and tablet lorazepam 4 mg in divided doses. However, the patient became non-compliant and discontinued medication after a few days, once his condition improved. There is no history of psychotic or mood disorders as corroborated by the informant. His past history was insignificant for any psychiatric or seizure disorders. In addition to the ICD-10 diagnosis of alcohol dependence syndrome with episodic use (dipsomania), we also diagnosed him as having an alcohol-induced psychotic disorder due to his current presentation. As requested by the caregiver, we treated the patient as an outpatient with oral lorazepam up to 4 mg/day, and tablet olanzapine (10 mg once at night) was added for seven days as an extra measure, considering our inability to supervise him after discharge. Along with pharmacological management, the psychosocial intervention was done where brief intervention was attempted but primarily the caregiver explained the condition of the patient, the cause and the possible consequences of his alcohol consumption, and the need for regular follow-ups. A telephonic conversation was made after three days with the informant, and she reported that the patient had improved and had begun working. However, the patient was lost to follow-up, and as per the informant, after a period of seven days, he did not continue with the course of treatment. 

## Discussion

Even in the absence of observable physiological withdrawal syndrome, catatonia can be an atypical manifestation of alcohol withdrawal [3]. The conventional benzodiazepine treatment, an established therapeutic choice for both catatonia and alcohol withdrawal, effectively addresses alcohol withdrawal catatonia [3]. This case shows a recurrent pattern of catatonia during the withdrawal period. Catatonia is a neuropsychiatric illness that most frequently occurs in mood disorders but can also occur in neurologic, medical, and psychotic disorders. It is characterized by aberrant movements, behaviors, and withdrawal. The majority of catatonic episodes fall into one of three categories: excited, retarded, or malignant [4]. Patients affected by these episodes may experience times of withdrawal and periods of stimulation as their symptoms wax, wane, or change. These various subtypes are supported by a networked system that connects the cortex, basal ganglia, and thalamus and causes catatonic symptoms [5]. Catatonia can be misdiagnosed with serious conditions like neuro malignant syndrome, non-psychiatric stupor, encephalopathy, stroke, Parkinson’s disease, locked-in syndrome, malignant hyperthermia, non-convulsive status epileptics, etc. Therefore, ideally, a thorough check-up should be done to rule out such events [6].

Previously reported cases predominantly showed catatonic features either in the presence of other substances or a comorbid condition. Two cases highlighted catatonic features without any comorbid condition, both originating from the Indian subcontinent [7,8]. Dutt et al. revealed the importance of adopting a systematic approach toward management, hence making a treatment protocol to avoid delays or inadequacy in the process [8]. Catatonia can hinder therapy, confuse diagnosis, and be potentially lethal if left untreated; therefore, recognizing and treating it can be crucial in both psychiatric and medical care. Early recognition, a thorough diagnostic approach, and an interdisciplinary treatment strategy are paramount to achieving successful outcomes in patients with alcohol withdrawal catatonia. This case report underscores the importance of a comprehensive understanding of alcohol withdrawal catatonia to optimize patient care and outcomes.

## Conclusions

Catatonia caused by alcohol withdrawal is an uncommon occurrence in clinical settings, especially when there are no coexisting conditions. Only two cases have been documented in India, which indicates how uncommon the syndrome is. Our report not only underscores the need for heightened awareness among healthcare providers but also emphasizes the importance of a multidisciplinary approach encompassing psychiatry, neurology, and addiction medicine in the management of such cases. By shedding light on this intriguing clinical scenario, we endeavor to enhance clinical knowledge and promote better outcomes for individuals grappling with alcohol use disorder and its myriad complications, including recurrent alcohol withdrawal catatonia.
